# Compensatory changes in CYP expression in three different toxicology mouse models: CAR-null, Cyp3a-null, and Cyp2b9/10/13-null mice

**DOI:** 10.1371/journal.pone.0174355

**Published:** 2017-03-28

**Authors:** Ramiya Kumar, Linda C. Mota, Elizabeth J. Litoff, John P. Rooney, W. Tyler Boswell, Elliott Courter, Charles M. Henderson, Juan P. Hernandez, J. Christopher Corton, David D. Moore, William S. Baldwin

**Affiliations:** 1 Biological Sciences, Clemson University, Clemson, SC, United States of America; 2 Environmental Toxicology, Clemson University, Pendleton, SC, United States of America; 3 NHEERL, US-EPA, Research Triangle Park, NC, United States of America; 4 Molecular and Cellular Biology, Baylor College of Medicine, Houston, TX, United States of America; INRA, FRANCE

## Abstract

Targeted mutant models are common in mechanistic toxicology experiments investigating the absorption, metabolism, distribution, or elimination (ADME) of chemicals from individuals. Key models include those for xenosensing transcription factors and cytochrome P450s (CYP). Here we investigated changes in transcript levels, protein expression, and steroid hydroxylation of several xenobiotic detoxifying CYPs in constitutive androstane receptor (CAR)-null and two CYP-null mouse models that have subfamily members regulated by CAR; the Cyp3a-null and a newly described Cyp2b9/10/13-null mouse model. Compensatory changes in CYP expression that occur in these models may also occur in polymorphic humans, or may complicate interpretation of ADME studies performed using these models. The loss of CAR causes significant changes in several CYPs probably due to loss of CAR-mediated constitutive regulation of these CYPs. Expression and activity changes include significant repression of Cyp2a and Cyp2b members with corresponding drops in 6α- and 16β-testosterone hydroxylase activity. Further, the ratio of 6α-/15α-hydroxylase activity, a biomarker of sexual dimorphism in the liver, indicates masculinization of female CAR-null mice, suggesting a role for CAR in the regulation of sexually dimorphic liver CYP profiles. The loss of Cyp3a causes fewer changes than CAR. Nevertheless, there are compensatory changes including gender-specific increases in Cyp2a and Cyp2b. Cyp2a and Cyp2b were down-regulated in CAR-null mice, suggesting activation of CAR and potentially PXR following loss of the Cyp3a members. However, the loss of Cyp2b causes few changes in hepatic CYP transcript levels and almost no significant compensatory changes in protein expression or activity with the possible exception of 6α-hydroxylase activity. This lack of a compensatory response in the Cyp2b9/10/13-null mice is probably due to low CYP2B hepatic expression, especially in male mice. Overall, compensatory and regulatory CYP changes followed the order CAR-null > Cyp3a-null > Cyp2b-null mice.

## Introduction

Nullizygous mouse models have become commonplace in toxicology research [1, 2], especially the use of xenobiotic receptor and Cyp subfamily-null mice [3–6]. These models are widely used in the study of the metabolism and distribution of pharmaceuticals and hazardous environmental chemicals [4–6]. To properly interpret the data observed, especially within absorption, distribution, metabolism, and excretion (ADME) studies, it is critical to have an understanding of the compensatory changes in cytochrome P450 (CYP) expression that occurs in these mouse models. The purpose of this study is in part to evaluate changes that occur in constitutive androstane receptor (CAR)-null, Cyp3a-null, and the newly developed Cyp2b9/10/13-null mouse models, estimate the impact that compensatory changes may have on xenobiotic metabolism, and interpret the basis for these changes.

The constitutive androstane receptor (CAR; NR1I3) is a xenobiotic sensor activated either directly by ligand binding such as 1,4-bis [2-(3,5-dichloropyridoxy)] benzene (TCPOBOP) [7] or indirectly in which the chemical of interest induces nuclear translocation through changes in phosphorylation status such as phenobarbital [8–10]. Modulators of CAR activity include environmental pollutants, pharmaceuticals, natural products, and endogenous chemicals such as steroids, bile acids, and fatty acids [3, 11–13]. CAR activation leads to increased transcription of genes involved in phase I-III detoxication, including the cytochrome P450s (CYP) with greater CYP2B6 induction than CYP3A4 or CYP2C9 induction [14, 15]. We have observed compensatory changes in CYP expression in CAR-null mice on the B6/SV129 background [16]. Here we take a more comprehensive look at compensatory changes in CAR-null mice, but on the B6 background.

CYP3A is the most predominant CYP in the liver encompassing 30–40% of the total hepatic CYP content and metabolizing more than 60% of the drugs available on the market [17]. In addition to the metabolism of numerous pharmaceuticals and environmental pollutants [3, 18]), CYP3A metabolizes endogenous molecules such as lithocholic acid [19], arachidonic acid [20] and steroid hormones [21]. Recently, knocking out *Cyp3a* was shown to increase Cyp2c-mediated metabolism of midazolam [22], potentially due to activation of the pregnane X receptor (PXR). Because Cyp3a is the predominant hepatic CYP and of such importance in toxicology, it is likely that loss causes compensatory mechanisms that alter the metabolism of endogenous and exogenous substances. In some cases these alterations may not be (at least in part) due to loss of Cyp3a, but instead increases in the production of other metabolites produced through the induction of CYPs in subfamilies 2a, 2b, and 2c.

There are currently two different Cyp3a knockout mouse models; not including humanized models. In the model produced by Van Herwaarden et al [6] on an FVB background, the Cyp3a members clustered in a 0.8Mb region of chromosome 5 were eliminated by Cre-lox, while Cyp3a13 located 7 Mb centromeric to the cluster was deleted by traditional targeting methods [6]. Another model produced on the C57Bl6 (B6) background, eliminated all of the Cyp3a members on the chromosome 5 cluster by Cre-lox, but did not eliminate Cyp3a13 [5, 23]. For the purposes of the present study this model is more attractive, because responses in this mouse can be compared to other mouse strains on the B6 background.

CYP2B is probably the least studied of the hepatic detoxication CYPs in families 1–3 because it was traditionally considered to have <1% of total hepatic CYP expression and in turn was called the overlooked or forgotten CYP [24]. However, recent studies using more sensitive probes and inhibitors have shown that CYP2B6 constitutes 2–10% of the total CYP expressed in the liver. It is estimated that CYP2B6 metabolizes approximately 25% of drugs available on the market [24] such as efavirenz [25], bupropion [26] and cyclophosphamide [27]. In addition, CYP2B metabolizes environmental pollutants such as nonylphenol [28], parathion [29] and polychlorinated biphenyls [30], and endogenous molecules such as testosterone [31], arachidonic acid [32, 33], linoleic acid [12] and epoxyeicosatrienoic acid [34].

We produce and describe the first exclusive Cyp2b-null mouse model in this manuscript. There are other models that lack Cyp2b or Cyp2b activity. These include P450 oxidoreductase-null mice (HRN or POR-null) that lack all CYP activity because they lack this crucial cofactor [35, 36], and the Cyp2a(4/5)bgs-null mouse model that lacks a 1.2 megabase region of chromosome 7 containing Cyp2a4, 2a5, 2b9, 2b10, 2b13, 2b19, 2b23, 2g1, 2s1, Nalp9a, Nalp9c, Nalp4a, Vmn1r185, and Vmn14184 [37]. This mouse model lacks all of the Cyp2b members, but also lacks other Cyp2 members (2a4, 2a5, 2g1, 2s1) as well as five non-CYP genes found between the two Cyp clusters on chromosome 7 [38]. Our new mouse model lacks three of the five Cyp2b members, 2b9, 2b10, and 2b13, which are the primary hepatic Cyp2b’s found in tandem repeat [38–40]. There are six genes between the Cyp2b9/10/13 cluster and Cyp2b19 and Cyp2b23 (S1 Fig). We did not delete Cyp2b19, which is primarily expressed in skin [34] and testes [41], or Cyp2b23, which until recently was not known to be expressed [41]. Recent work suggests Cyp2b23 is expressed briefly at very low levels in the livers of young mice [39, 42].

Many of the xenobiotic detoxifying CYPs are expressed in a sexually dimorphic manner [43–45]. Murine male predominant hepatic CYPs include Cyp2d9 and 4a12 [44, 46], which are not regulated by CAR. Murine female predominant hepatic CYPs include Cyps 2a4, 2b9, 3a41, 2c40 and 3a44 [43, 44, 46–48], of which several are regulated by CAR [16]. Furthermore, CAR demonstrates greater expression [49] and activity in females than males [50]. This may be due to increased regulation of CAR by hepatocyte nuclear factor 4α (HNF4α) in females [51], estrogen activation of CAR [52], androgen inhibition of CAR [11, 53], or a combination of these factors. Taken together, this data indicates that CAR has greater activity in female mice and therefore maintains basal expression of several CYPs in a sexually dimorphic fashion. Therefore, changes in the basal expression of several CYPs, including several female predominant CYPs will be compared between wild-type and CAR/CYP-null mice.

The overall purpose of this manuscript is to compare and evaluate changes in CYP gene expression, protein expression, and enzyme activity in three toxicology knockout models; CAR, Cyp3a-null, and Cyp2b9/10/13-null mice. CAR is a key regulator of Cyp2b and Cyp3a expression and therefore these CYP models are reasonable models to investigate in order to discern how they are altered in comparison to CAR-null mice.

## Materials and methods

### Mice

All studies were carried out according to NIH guidelines for the humane use of research animals and were pre-approved by the Baylor College of Medicine or Clemson University Animal Care and Use Committee. Mice are on a C57/Bl6 (B6) background, provided water and food *ad libitum*, and between 8–11 weeks old at the time of euthanasia. Mice were euthanized by carbon dioxide asphyxiation followed by bilateral thoracotomy. While each mouse is on a B6-background, each B6 mouse is from a different source. CAR-null mice [7, 54] and their B6 wild-type (WT) controls (B6) were housed at Baylor College of Medicine (BCM) on the B6 background bred at BCM. Cyp3a-null mice [5] and their respective B6-WT controls were obtained from Taconic (Hudson, NY USA). These mice lack the *Cyp3a57*, *Cyp3a16*, *Cyp3a41*, *Cyp3a44*, *Cyp3a11*, *Cyp3a25*, and *Cyp3a59* genes all located within approximately 0.8Mb of each other in a tandem repeat region on chromosome 5, but still contain the *Cyp3a13* gene located 7 Mb centromeric from the cluster on chromosome 5 [5].

**Fig 1:**
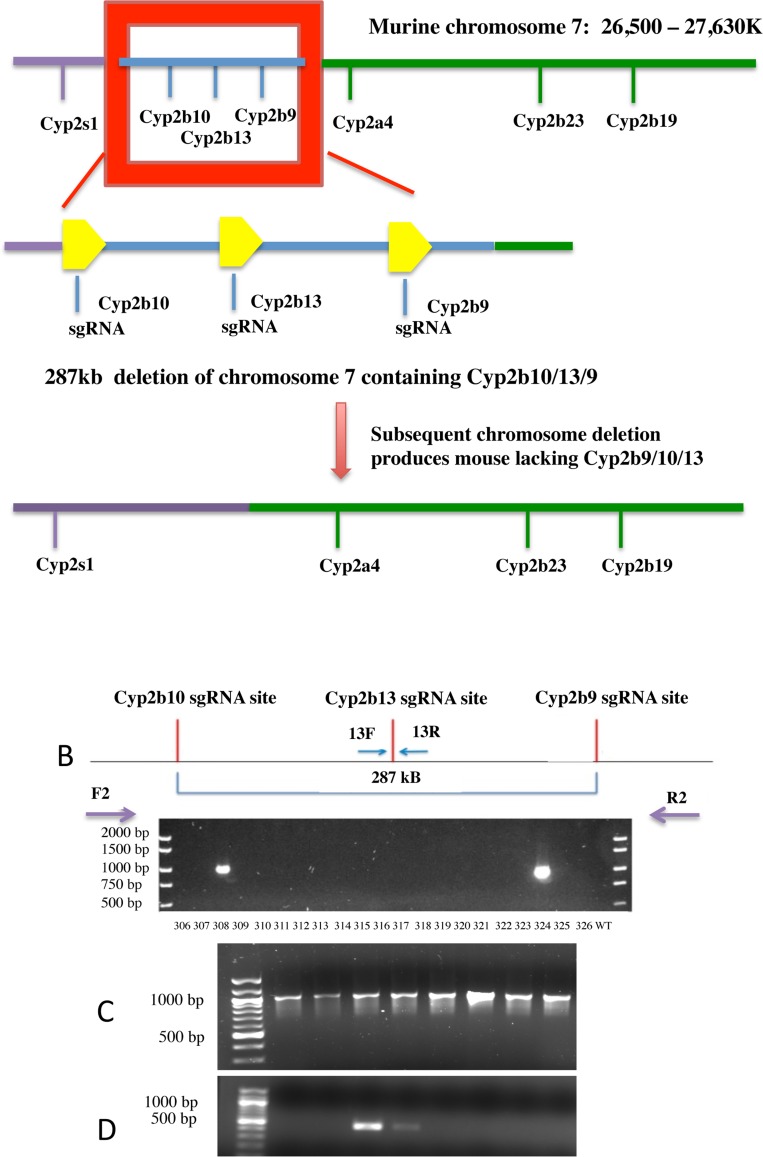
Construction of Cyp2b9/10/13-null mice. **(A)** Cyp2b9/10/13-null mice were produced using Crispr/Cas9 with sgRNA target sites for all three genes. A 287 kb deletion mutant was produced that lacks these three Cyp2b genes found in tandem repeat on chromosome 7. **(B)** Two mice heterozygote for a chromosome deletion lacking the three Cyp2b genes (Cyp2b10, 2b13, 2b9) in tandem repeat were produced. PCR confirmation of the 287kb deletion from the first null mice produced is shown in lanes 4 and 21 using the F2/R2 primer combination that produces a 1066 bp fragment. **(C)** Subsequent breeding produced mice lacking Cyp2b9/10/13 as demonstrated by the presence of the 1066 bp PCR product. **(D)** Heterozygotes were discerned from homozygotes by PCR of Cyp2b13 indicating a heterozygote.

Cyp2b9/10/13-null mice were produced using the Crispr/Cas9 system. Corresponding WT controls (B6) were obtained from The Jackson Laboratory (Bar Harbor, ME) and used to produce the Cyp2b9/10/13-null mice. Cyp2b9, Cyp2b10, and Cyp2b13 are the three Cyp2b members primarily expressed in the liver (Cyp2b9/10/13) and found in tandem repeat (S1 Fig)[38–40]. Each of the three hepatic genes was targeted (Fig 1). Cas9 mRNA from *Streptococcus pyogenes* and a 20nt guide sequence that was specific to the target site with an 83nt scaffold sequence, which was common to all the sgRNAs was injected into the cytoplasm of the mouse blastocyst [55]. The scaffold sequence was guuuuagagcuagaaauagcaaguuaaaauaaggcuaguccguuaucaacuugaaaaaguggcaccgagucggugcuuuuuuu. The Cyp2b10 guide sequence was: uguggaggagcggauucagg(AGG). The Cyp2b13 guide sequence was: (CCC)ugcaagagguuccccaagag, and the Cyp2b9 guide sequence was: acattgatacctaccttctg(AGG). The protospacer adjacent motif (PAM) is shown in parenthesis. The incorporation efficiency at each site in vitro was Cyp2b10, 47.6%, Cyp2b13, 33.3%, and Cyp2b9, 33.3%. The resultant injection of more than 100 embryos produced two mice with a 287 kB deletion lacking all three hepatic Cyp2b members found in tandem repeat. Each mouse was genotyped to ensure the presence of the knockout using the F2/R2 primer set (F2: 5’-gccagggtcagcatattcaccaa-3’ and R2: 5’-gcacagacatcatgaggttctggtg-3’) that produces a 1066 bp band in triple-gene knockout mice. To ensure we are not working with heterozygotes, we also genotyped for the presence of Cyp2b13 (F: 5’-cagactcttgttagaccggaccat-3’ and R: 5’-ccccaaggaataaaattctacatg-3’) (Fig 1).

### Sample preparation

Eight to eleven-week old B6, CAR-null, and Cyp2b9/10/13-null male and female mice (n = 4–6) were euthanized by CO_2_ asphyxiation. Livers were excised and diced into several pieces and snap frozen for RNA extraction or microsome preparation and then stored at -80^°^C. Eight to ten-week old Cyp3a-null and corresponding B6 controls from Taconic were euthanized at Taconic Biosciences (Hudson, NY USA), the livers were snap frozen and shipped on dry ice to Clemson. RNA was extracted from a little less than half of the liver using the Bio-Rad spin columns with DNAse (Bio-Rad, Hercules, CA USA) according to the manufacturer’s instructions. RNA concentrations were determined spectrophotometrically at 260/280 nm (Molecular Devices, Ramsey, MN USA). Reverse transcription was performed to make cDNA using 200 units MMLV-RT, a 10 mM dNTP mixture, and 0.05 mg random hexamers (Promega Corporation, Madison, WI USA). For microsome and cytosol preparation, approximately half of the liver was individually homogenized with a Dounce Homogenizer and protein fractions were prepared as described previously [56]. Protein concentrations were determined with the Bio-Rad protein assay (Bio-Rad) according to the manufacturer’s instructions.

### Quantitative Real-time Polymerase Chain Reaction (QPCR)

Quantitative real-time PCR (qPCR) was performed using primers for specific isoforms to Cyp2a, Cyp2b, Cyp2c, and Cyp3a subfamily members, or 18S as the housekeeping gene. All the qPCR primers were previously published [38, 43]. Samples were diluted 1:10 and amplifications of the standard curve performed in triplicate using a 96-well IQ^TM^ Real-Time PCR detection system (Bio-Rad) with 0.25X RT^2^ SybrGreen (Qiagen Frederick, MD USA) as the fluorescent double strand intercalating dye to quantify gene expression as described previously using Muller’s equation to determine relative quantities of each CYP [57, 58].

### Western blots

Western Blots were performed on 30 μg of microsomal protein to measure CYP levels. Proteins were separated by polyacrylamide gel electrophoresis (SDS-PAGE) in a 10% gel, and transferred to 0.45 μm nitrocellulose (Bio-Rad) where the blot was blocked using 1% skim milk/0.1% Tween 20 dissolved in phosphate buffered saline. Pre-stained protein standards (Bio-Rad) were used as molecular weight markers. Primary antibodies were obtained from a variety of sources. Rabbit anti-mouse Cyp2b10 antibody was produced by our laboratory and previously characterized [4, 59]. Rabbit anti-rat CYP3A1 and rabbit anti-human CYP2C8/9/19 were obtained from Chemicon International (Temecula, CA USA). Mouse anti-human CYP2A6 was originally obtained from Gentest™ Corporation (San Jose, CA USA) and used with the CAR-null and Cyp3a-null mice. CYP2A6 antibody was later obtained from Thermo-Fisher (Rockford, IL) for use with the Cyp2b9/10/13-null mice when the original stock from Gentest was no longer available. Rabbit anti-mouse β-actin (Sigma Aldrich, St. Louis, MO USA) was used to ensure equal loading of samples. Goat anti-rabbit IgG (Bio-Rad) alkaline-phosphatase coupled secondary antibodies were used for recognizing CYP2A6, Cyp2b10, CYP3A1, and CYP2C8/9/19 primary antibodies. Goat anti-mouse (Bio-Rad) IgG were used to recognize the β-Actin primary antibodies. Primary antibodies were diluted 1:1000, and secondary antibodies were diluted 1:500. Bands were visualized using a chemiluminescent kit according to the manufacturer’s directions (Bio-rad). Chemiluminescence was quantified on a Chemi-Doc system with Quantity One software (Bio-Rad). Western blot results regarding specific CYP protein data are referred to as subfamilies (i.e. CYP3A) instead of a specific protein because the antibodies most likely recognize several different subfamily members [38, 60].

### Testosterone hydroxylase assays

Testosterone hydroxylase assays were used to measure CYP activity as previously described [43]. [4-^14^C]Testosterone (Perkin-Elmer, Waltham, MA) was used to visualize testosterone metabolites separated by thin-layer chromatography and quantify. testosterone metabolites with a LS5801 liquid scintillation counter (Beckman, Fullerton, CA USA).

### Microarrays

There were 3 or 4 biological replicates used for each of the genotype-sex groups (GSE90614). Liver RNA was isolated by mechanical disruption followed by RNAzol and was further purified using silica membrane spin columns (RNeasy®, Qiagen, Valencia, CA). RNA integrity was assessed by the RNA 6000 LabChip® kit using a 2100 Bioanalyzer (Agilent Technologies, Palo Alto, CA). Gene expression in the livers of the mice was evaluated using Affymetrix mouse 430PM arrays. Procedures for labeling, hybridization, washing and scanning were carried out according to the manufacturer's recommendations. Gene expression results were analyzed in Partek Genomic Suite by standard methods. Briefly, .cel files were imported and normalized by Robust Multichip Average (RMA). Differentially expressed genes (DEGs) were determined by ANOVA with a false discovery rate of 0.05, and a fold change cutoff of +/- 1.2 fold.

### Assembly of Cyp expression data from microarray studies carried out in CAR-null mice

Comparisons of publicly available gene expression profiles were conducted using the meta-analysis function of the Illumina BaseSpace Correlation Engine. The meta-analysis function allows for specified gene expression profiles (called “biosets” within Correlation engine) to be examined for gene expression changes. We compared biosets from CAR-null mice (GSE40120) and from 28 and 91-day old CAR/PXR-null mice (GSE60684), which most closely approximate the ages of the mice used in this study, and then filtered gene expression data to investigate changes in expression of Cyp family members.

### Statistical analysis

Statistical tests were performed with GraphPad Prism software 6.0 (La Jolla, CA USA). ANOVA was used to compare three or more treatment groups followed by Fisher’s PLSD as the *post-hoc* test, and a p-value of ≤ 0.05 was regarded as significantly different from control values.

## Results and discussion

### CAR-null mice

CAR regulates the expression of Cyp2a, Cyp2b, and Cyp3a subfamily members [3]. We examined the expression of these subfamily members in part based on the work of ourselves and others that indicates HNF4α regulates CAR expression with HNF4α > CAR > PXR regulation of constitutive *Cyp* expression [16, 61]. Thus, we examined the expression of CYPs previously shown to be constitutively regulated by HNF4α [44] and hypothesized that CAR-null mice would show changes in constitutive CYP gene expression, corresponding protein expression and enzyme activity. HNF4**α** and to a lesser extent CAR are crucial transcription factors in the sexually dimorphic expression of hepatic CYPs, including Cyp2a4, Cyp2b9, Cyp2b10, Cyp2b13, Cyp3a41, and Cyp3a44 [16, 44]. CAR-null female mice show significant down-regulation of *Cyp2b9*, *Cyp2b10*, *Cyp2b13*, and *Cyp3a11* compared to WT-B6 mice. Interestingly, Cyp3a11 showed slight female predominance, about 1.7X, in two of our three studies in the WT (B6) mice (Tables 1–3). Previous work with B6/SV129 mice indicated that Cyp3a11 is female predominant [16]; however studies with FVB/NJ mice indicate that Cyp3a11 expression is gender neutral [43].

**Table 1:** Compensatory changes in CYP gene expression in CAR-null mice.

	MALES		FEMALES	
**Gene**	WT#	CAR-null	WT	CAR-null
Cyp2a4	1.00 ± 0.241	5.276 ± 0.904	13.983 ± 0.762^c^	15.241 ± 3.940^b^
Cyp2b9	1.00 ± 0.353	0.424 ± 0.096	18.35 + 2.898^c^	0.151 ± 0.063^b^
Cyp2b10	1.00 ± 0.692	0.196 ± 0.027	10.186 ± 2.249^c^	0.054 ± 0.011^b^
Cyp2b13	1.00 ± 0.312	0.021 ± 0.006	4.139 ± 0.798^c^	0.009 ± 0.004^b^
Cyp2c29	1.00 ± 0.251	0.395 ± 0.388	0.306 ± 0.091	0.188 ± 0.082
Cyp2c40	1.00 ± 0.950	0.805 ± 0.753	6.992 ± 0.551^c^	3.174 ± 0.936^b^
Cyp3a11	1.00 ± 0.341	0.461 ± 0.085	1.805 ± 0.283^c^	0.904 ± 0.176^b^
Cyp3a41	1.00 ± 0.475	430.0 + 405.8	81.01 + 33.43^c^	442.0 + 178.9

#Data are presented as relative mean ± SEM. Statistical significance determined by ANOVA followed by Fisher’s LSD as the post-hoc test (n = 5–6).

‘b’ indicates WT females different than CAR-null females.

‘c’ indicates WT males different than WT females.

Letter with no asterisk indicates a p-value < 0.05

**Table 2:** Compensatory changes in CYP gene expression in Cyp3a-null mice.

	MALES		FEMALES	
**Gene**	WT#	Cyp3a-null	WT	Cyp3a-null
Cyp2a4	1.00 ± 0.328	47.957 ± 16.267^a^	14.326± 0.708	70.06 ± 20.30^b^
Cyp2b9	1.00 ± 0.321	7.743± 7.319	117.96±7.899^c^***	115.134±20.868^d^***
Cyp2b10	1.00 ± 0.310	6.247 ± 4.831	9.7± 1.447^c^	13.435 ± 2.435
Cyp2c29	1.00 ± 0.457	29.431±15.874	5.193 ± 2.213	23.586 ±7.049
Cyp2c40	1.00 ± 0.413	25.913±15.709	21.722±12.568	38.772 ± 4.732
Cyp3a11	1.00 ± 0.168	0.000^a^*	1.68 ± 0.336^c^	0.000^b^***
Cyp3a13	1.00 ± 0.371	26.143± 9.424^a^	6.294±1.12	28.882 ± 8.366^b^
Cyp3a25	1.00 ± 0.247	0.002 ± 0.001^a^**	0.75 ± 0.121	0.001 ± 0.001^b^*
Cyp3a41	1.00 ± 0.131	0.000	14.138±3.967^c^**	0.000^b^**

^**#**^Data represented as mean +/- SEM (n = 4). Statistical significance determined by one-way ANOVA followed by LSD as the post-hoc test.

‘a’ indicates WT males different than Cyp3a-null males.

‘b’ indicates WT females different than Cyp3a-null females.

‘c’ indicates WT males different than WT females.

‘d’ indicates Cyp3a-null males different than Cyp3a-null females.

Letter with no asterisk indicates a p-value < 0.05 and

* indicates a p-value < 0.01,

** indicates a p-value <0.001,

***indicates a p-value <0.00001.

**Table 3:** Compensatory changes in CYP gene expression in Cyp2b9/10/13-null mice.

	MALES		FEMALES	
**Gene**	WT#	Cyp2b9/10/13-null	WT	Cyp2b9/10/13-null
Cyp2a4	1.00 ± 0.187	5.162 ± 3.948	18.864±4.066^c^*	7.217 ± 2.294^b^
Cyp2b9	1.00 ± 0.692	0.054 ± 0.018	3.007 ± 0.897^c^	0.047 ± 0.020^b^*
Cyp2b10	1.00 ± 0.655	0.041 ± 0.014	2.274 ± 0.678	0.035 ± 0.015^b^*
Cyp2b13	1.00 ± 0.709	0.059 ± 0.048	36.189 ± 7.478^c^***	0.082± 0.078^b^**
Cyp2c29	1.00 ± 0.091	2.277 ± 0.760	0.628 ± 0.123	1.472 ± 0.792
Cyp2c40	1.00 ± 0.123	0.911 ± 0.636	5.202 ± 2.169^c^	1.620 ± 0.846^b^
Cyp3a11	1.00 ± 0.124	0.790 ± 0.173	0.828 ± 0.101	0.524 ± 0.045
Cyp3a13	1.00 ± 0.170	0.551 ± 0.114	1.088 ± 0.121	0.536 ± 0.077^b^
Cyp3a25	1.00 ± 0.346	0.983 ± 0.350	0.991 ± 0.083	0.515 ± 0.126
Cyp3a41	1.00 ± 0.756	0.112 ± 0.107	19.459 ± 5.931^c^*	11.168 ± 1.627^d^

^#^Data represented as mean +/- SEM (n = 4). Statistical significance determined by one-way ANOVA followed by LSD as the post-hoc test.

‘b’ indicates WT females different than Cyp2b9/10/13-null females.

‘c’ indicates WT males different than WT females.

‘d’ indicates Cyp2b9/10/13-null males different than Cyp2b9/10/13-null females.

Letter with no asterisk indicates a p-value < 0.05 and

* indicates a p-value < 0.01,

** indicates a p-value <0.001,

***indicates a p-value <0.00001

CAR-null females show nearly a complete loss of Cyp2b13 and 6- and 19-fold decrease in Cyp2b9 and Cyp2b10, respectively. CAR-null males show a similar trend with respect to these Cyp2b members, although the data were not significant (Table 1), most likely because most Cyp2b members show lower expression in males [3]. Cyp2c40 expression decreased nearly 2-fold in CAR-null female mice, but these mice still showed higher expression of this female predominant CYP than male mice. Of the female predominant CYPs examined only Cyp2a4 showed increased expression in CAR-null mice (Table 1). This result suggests that CAR negatively regulates Cyp2a4 expression. Results from HNF4a-null mice indicate that Cyp2a4 is also negatively regulated by HNF4α [44]. Given HNF4α’s role in regulating CAR [61], it is possible that HNF4α in part regulates Cyp2a4 by regulating CAR expression and activity.

**Fig 2:**
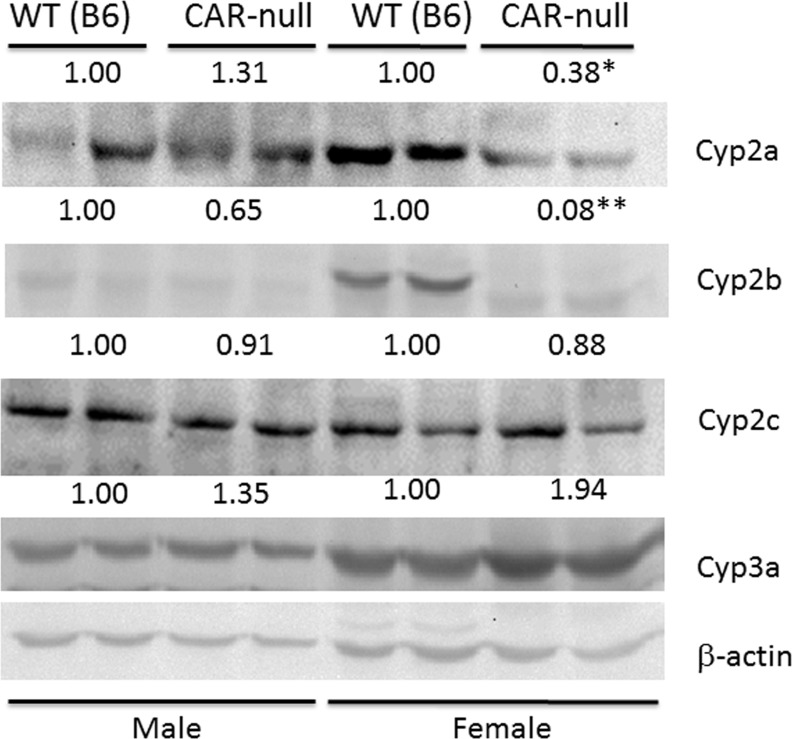
Compensatory changes in CYP protein expression in CAR-null mice. Western blots of male and female CAR-null mice show significant changes in CYP expression relative to their WT counterparts. Results are expressed as relative mean of the WT compared to CAR-null mice of the same sex. Statistical differences were determined by Student’s t-tests (n = 2) with * (p < 0.05) ** (p < 0.01) indicating significant differences.

**Fig 3:**
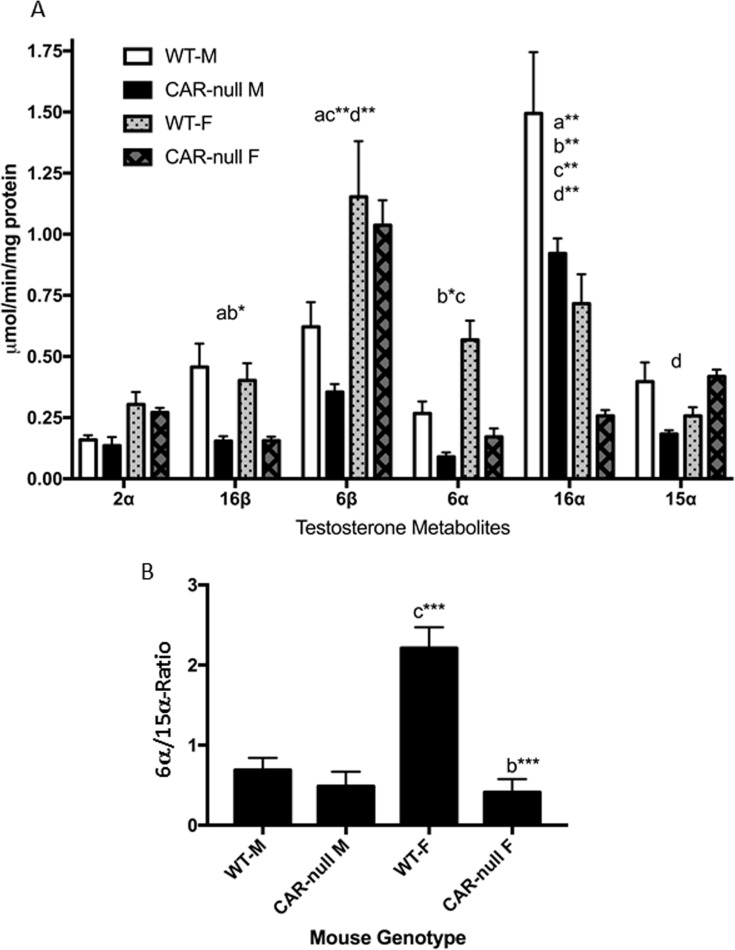
Testosterone hydroxylation is perturbed in CAR-null mice in a gender-specific manner. **(A)** Testosterone hydroxylation was determined in male and female WT and CAR-null mice as described in the Materials and Methods. Data are presented as mean specific activity (μmol/min/mg protein) ± SEM (n = 5). **(B)** Ratio of 6α/15α-hydroxytestosterone as a biomarker of CYP sexual dimorphism in the liver. An ^a^indicates a significant difference between WT male and Cyp2b9/10/13-null male mice, ^b^indicates a significant difference between WT female and Cyp2b9/10/13-null female mice, ^c^indicates a significant difference between male and female WT mice and ^d^indicates a significant difference between the male and female Cyp2b9/10/13-null mice. Statistical differences were determined by two-way ANOVA followed by Fisher’s LSD as the post-hoc test in (A) and one-way ANOVA followed by Fisher’s LSD in (B). A letter without an asterisk indicates a significance of p < 0.05, asterisk indicate significance of *p<0.01, **p<0.001, and *** p<0.0001, respectively.

Protein expression and testosterone hydroxylase activity generally corresponded well to the qPCR results. Cyp2b9, 2b10, and 2b13 were all repressed in CAR-null female mice, CYP2B protein concentrations were significantly reduced as determined by Western blots (Fig 2) and 16α– and 16β-hydroxylase activities (Fig 3), both of which are associated with CYP2B or CYP2B induction were repressed as expected [21, 60]. In addition, Cyp3a mRNA expression, CYP3A protein expression, and 6β-hydroxylase activity were all higher in WT females than WT males (Table 1; Figs 2 and 3). However, 6β-hydroxylase activity in CAR-null males compared to WT males were not consistent with protein expression, but comparable to the qPCR data. In addition, CYP2A protein was significantly down-regulated in CAR-null females (Fig 2), while Cyp2a4 mRNA was increased (Table 1). There was no significant difference in the Cyp2a-mediated, 15**α** -hydroxylase activity between WT and the corresponding CAR-null mice of the same sex. Overall, these minor discrepancies may be due to preferential antibody recognition of specific Cyp3a or 2a subfamily members such as Cyp3a13, 41, and 44, or Cyp2a5.

Sexual dimorphism is observed in WT mice in the production of 6β-, 6α-, and 16α-OH testosterone (Fig 3). Sexual dimorphic differences in testosterone hydroxylation are also clear in the CAR-null mice. For example, we observed significant induction of 15α-OH testosterone in CAR-null females, and a drop in 6β-OH testosterone production in CAR-null males compared to WT males (Fig 3). Therefore, we examined the 6α/15α-OH testosterone ratio, which is much greater in females than males, controlled by androgen status, and considered a biomarker of androgen disruption in mice [62]. The 6α/15α-OH testosterone ratio is 3.2-fold higher in WT females than WT males. However, 6α/15α-OH testosterone ratio is 5.1-fold higher in WT females than CAR-null females and in turn the 6α/15α-OH testosterone ratio is 1.2-fold higher in CAR-null males than CAR-null females; the opposite direction of what is expected. Thus, the CAR-null females have a lower 6α/15α-OH testosterone ratio than WT males because of masculinization of CYP profiles in the liver in CAR-null females. However, the CAR-null females show no differences in liver concentrations of testosterone (S2 Fig).

Overall, the masculinization of hepatic testosterone metabolism profiles reflects the systematic loss of female predominant CYPs in the CAR-null mice (Table 1). CAR may regulate sexual dimorphism in the liver in mice through androgen inhibition in males as several different androgens are CAR inverse agonists [53], including androgens used as performance enhancing drugs [11]. Interestingly, PXR-null mice also show sexual dimorphic effects by promoting estrogenic activity due to the loss of sulfotransferase-mediated estrogen metabolism through activated PXR [63, 64]. However, more likely sexually dimorphic differences or loss of sexual dimorphism is directly due to the loss of CAR and its role in regulating female predominant CYPs in conjunction with HNF4α [16, 44, 61].

Sexual dimorphism of hepatic CYPs is primarily regulated by the periodization of growth hormone release that regulates Stat5b [45, 65–67]. Other transcription factors regulated in a sexually dimorphic fashion that in turn regulate sexually dimorphism include HNF4α, FoxA2, and CAR [16, 44, 68, 69]. For example, FoxA2 promotes the expression of the female specific hepatic CYP, Cyp2b9; HNF4α positively regulates the expression of several CYPs including Cyp2b10, Cyp2b13, Cyp3a41, and Cyp3a44 in females and negatively regulates Cyp2b9 and Cyp2a4 in males [44]; and CAR is thought to positively regulate Cyp2b13, Cyp2c29, and potentially Cyp2b10 [16]. Some of CAR’s sexually dimorphic activity may be direct, but some may also be due to HNF4α’s regulation of CAR [61]. Independent studies by Baldwin’s [16, 43] and Corton’s laboratories [67] have shown chemical activation of CAR can induce feminization of the liver. Taken together, masculinization of the liver does not necessarily involve testosterone.

The presence of microarray data from CAR-null mice in GEO allowed us to perform comparisons between our data and female CAR-null and WT mice (GSE40120), and male CAR/PXR double-null and WT mice [70](GSE60684) from previous studies. In all comparisons the expression of the *Car* gene was suppressed (~3-14-fold) in the null mice, as expected. *Cyp* genes increased in expression included *Cyp2a5*, *Cyp2c38*, *Cyp2c39*, *Cyp2g1*, *Cyp4a14*, *Cyp51*, and *Cyp7a1*. *Cyp* genes decreased in either CAR-null or CAR/PXR-null mice included *Cyp2a12*, *Cyp2b10*, *Cyp2b9*, *Cyp2c29*, *Cyp2c37*, *Cyp2c50*, *Cyp2c54*, *Cyp2c70*, *Cyp2u1*, *Cyp4a12a*, *Cyp4v3*, and *Cyp7b1*. *Cyp2c55* was increased in male CAR-null mice and suppressed in female CAR/PXR-null mice. Genes in the Cyp2c subfamily were differentially expressed in both directions. This may account for discrepancies between the qPCR and Western blots for Cyp2c, and it is also possible that a drop in 16α-hydroxylase activity is in part due to a drop in Cyp2c expression or Cyp2b expression.

Overall, CAR is a regulator of the hepatic *Cyp2b* genes and loss of CAR causes a considerable drop in Cyp2b expression. Furthermore, CAR may regulate sexual dimorphism in the liver as loss of CAR activity decreases the 6α/15α-OH testosterone ratio, a biomarker of masculinization of the liver [16, 62] that may also reflect an overall drop in CYP activity [61]. CAR expression is female predominant emphasizing the need to include both genders in drug trials and toxicant biotransformation studies [49, 71]. This is important as it provides data that may help us inform translational studies or physicians when prescribing personalized medicines. It may also reveal the mechanism behind specific chemical sensitivities in patients with low CAR or CYP activities.

### Cyp3a-null mice

**Fig 4:**
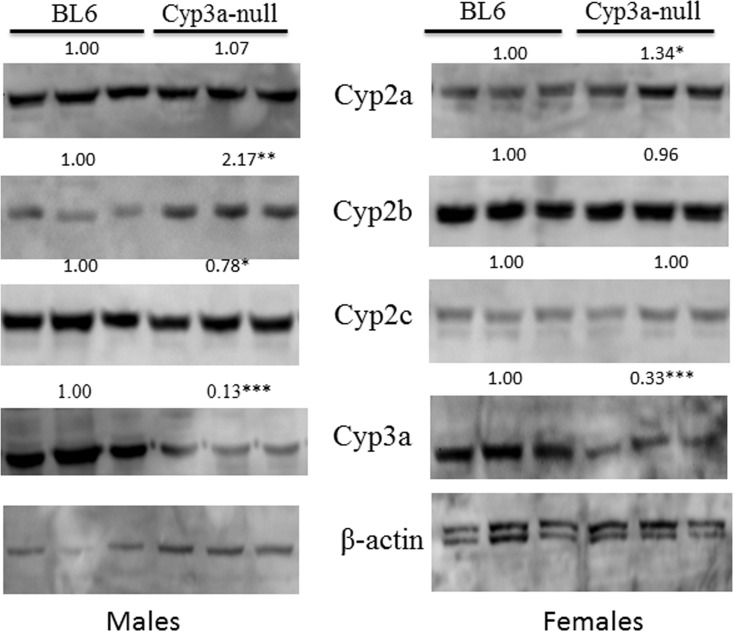
Compensatory changes in CYP protein expression in Cyp3a-null mice. Western blots of male and female Cyp3a-null mice show significant changes in CYP expression relative to their WT counterparts. Results are expressed as relative mean of the WT compared to CAR-null mice of the same sex. Statistical differences were determined by Student’s t-tests (n = 3) with * (p < 0.05) ** (p < 0.01) *** (p < 0.001) indicating significant differences.

CYP3A accounts for 30–40% of hepatic CYP expression [17]. We hypothesized that the loss of seven Cyp3a genes would perturb the hepatic P450 profile and lead to compensatory changes. One of the compensatory changes observed is an increase in Cyp3a13 expression (Table 2). Cyp3a13 is the only Cyp3a member not deleted, as it is 7Mb upstream from the 7-Cyp3a gene cluster [5]. Cyp3a13 is almost certainly the basis for recognition of CYP3A in Western blots (Fig 4) and the observation that 6β-hydroxylase activity only decreased 67% in males and 74% in females. Although other CYPs also produce 6β-hydroxytestosterone [72], data indicates that approximately 90% of 6β-hydroxytestosterone is produced by CYP3A enzymes in humans [72, 73].

Other CYPs also went through compensatory changes in the Cyp3a-null mice. qPCR data demonstrates significant induction (48- and 70-fold) of Cyp2a4 in Cyp3a-null female and male mice, respectively compared to their corresponding WT counterparts (Table 2). Western blots confirm the increase in CYP2A in females but not males (Fig 4). Cyp2a4 up-regulation is similar to the observations made in CAR-null mice, suggesting either a drop in CAR activity in Cyp3a-null mice or more likely an increase in PXR activity. CAR and PXR crosstalk and there is weak but insignificant induction of several other CAR/PXR regulated CYPs [4, 16, 74] including Cyp2b10, Cyp2c29, and Cyp2c40 (Table 2) with increased protein levels of CYP2B (Fig 4**)**. Therefore, we consider it more likely that PXR activity is increased potentially due to a lack of metabolism of a CYP3A metabolized endobiotic such as bile acids that in turn activate PXR [19, 75, 76].

**Fig 5:**
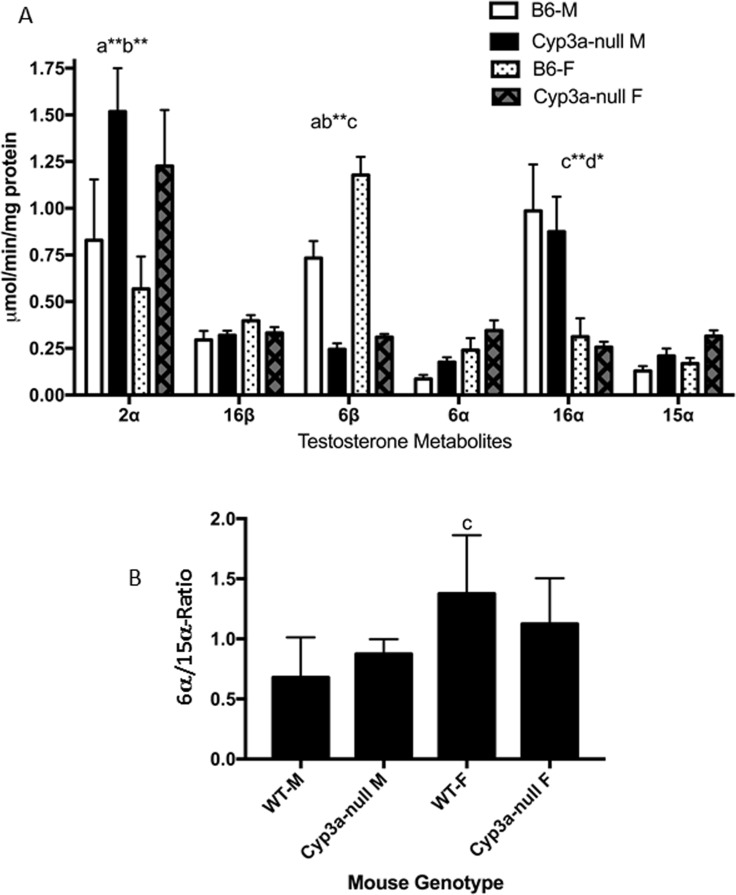
Changes in testosterone hydroxylation in Cy3a-null mice. **(A)** Testosterone hydroxylation was determined in male and female WT and Cyp3a-null mice as described in the Materials and Methods. Data are presented as mean specific activity (μmol/min/mg protein) ± SEM (n = 4). **(B)** Ratio of 6α/15α-hydroxytestosterone as a biomarker of CYP sexual dimorphism in the liver. An ^a^indicates a significant difference between WT male and Cyp2b9/10/13-null male mice, ^b^indicates a significant difference between WT female and Cyp2b9/10/13-null female mice, ^c^indicates a significant difference between male and female WT mice and ^d^indicates a significant difference between the male and female Cyp2b9/10/13-null mice. Statistical differences were determined by two-way ANOVA followed by Fisher’s LSD as the post-hoc test in (A) and one-way ANOVA followed by Fisher’s LSD in (B). A letter without an asterisk indicates a significance of p < 0.05, asterisk indicate significance of *p<0.01, **p<0.001, and *** p<0.0001, respectively.

Testosterone hydroxylase activity was greatly diminished at the 6β-position as expected because of the loss of CYP3A. Few other testosterone hydroxylase activities are perturbed significantly with the exception of 2α-hydroxylase activity (Fig 5). Hydroxylation of testosterone in the 2α-position is primarily considered a product of CYP2C. An increase in CYP2C protein was not measured; however, Cyp2c members were up to 29-fold higher by qPCR. This may be due to the CYP2C antibody preferentially recognizing CYP2C members not induced in the Cyp3a-null mouse model. CYP2C is increased in Cyp3a-null mice exposed to the PXR activator midazolam [22, 77], but CYP2C induction may be muted or lacking in untreated Cyp3a-null mice. Another possibility is that there are interactions between the hydroxylated products of testosterone. For example, 2α-hydroxylation of testosterone may be inhibited by 6β-hydroxytestosterone, and in turn the loss of CYP3A activity allows for increased 2α-hydroxylation. Ultimately, the data suggests the potential for compensatory CYP activity in the Cyp3a-null model complicating the interpretation of xenobiotic metabolism data. This observation is supported by previous work [22, 77].

6α- and 15α-hydroxylation were increased in Cyp3a-knockout mice, but not significantly despite a significant increase in Cyp2a4 mRNA and CYP2A protein in Cyp3a-null female mice compared to WT female mice (Fig 4; Table 2). These hydroxylase activities are mediated by sexually dimorphic CYP2A members in mice [47, 78]. Therefore, we examined the 6α/15α ratio. The 6α/15α-OH testosterone ratio is 2.03-fold higher in WT females than WT males; however the ratio drops to 1.29 when comparing Cyp3a-null females to Cyp3a-null males (not significantly different). The changes are not nearly as large as observed in the CAR-null mice, but the loss of significant sexual dimorphism suggests the potential for a small to moderate increase in hepatic masculinization of the females coupled with a small amount of feminization of the males. No differences in hepatic testosterone were observed (S2 Fig).

CYP3A metabolizes 50–60% of drugs available in the market [17] and is inhibited by fatty liver disease [79] and diabetes mellitus [80]. Because CYP3A plays such a prominent role in drug metabolism, changes in CYP3A expression and activity are crucial during the development of pharmaceuticals. Overall, the loss of CYP3A caused minor changes in the expression of the other CYPs examined with minimal changes in activity at least under untreated (pristine) conditions. There is minimal masculinization of liver testosterone hydroxylase activities; however, these changes are not as strong as observed in CAR-null mice. Taken together, Cyp3a-null mice may show compensatory metabolism of drugs by other CYPs that may compound the interpretation of the metabolism data; however, most of the lost CYP activity is directly attributable to the loss of CYP3A members (Fig 5).

### Cyp2b9/10/13-null mice

CYP2B metabolizes approximately 25% of drugs available in the market despite making up only 5–10% of the total CYPs expressed in the human liver [24]. We hypothesized that the lack of Cyp2b9, Cyp2b10 and Cy2b13 will lead to compensatory changes by altering CYP expression levels in the liver. In addition to a decrease in Cyp2b9, 10 and 13 gene expression, we also observed significant down regulation of Cyp2a4, Cyp2c40 and Cyp3a13 mRNA in Cyp2b9/10/13-null female mice compared to WT female mice (Table 3). However, significant changes were not observed in Cyp2b9/10/13-null male mice potentially because these Cyp2b subtypes, especially Cyp2b9 and Cyp2b13 are primarily expressed in female liver [16, 81].

**Fig 6:**
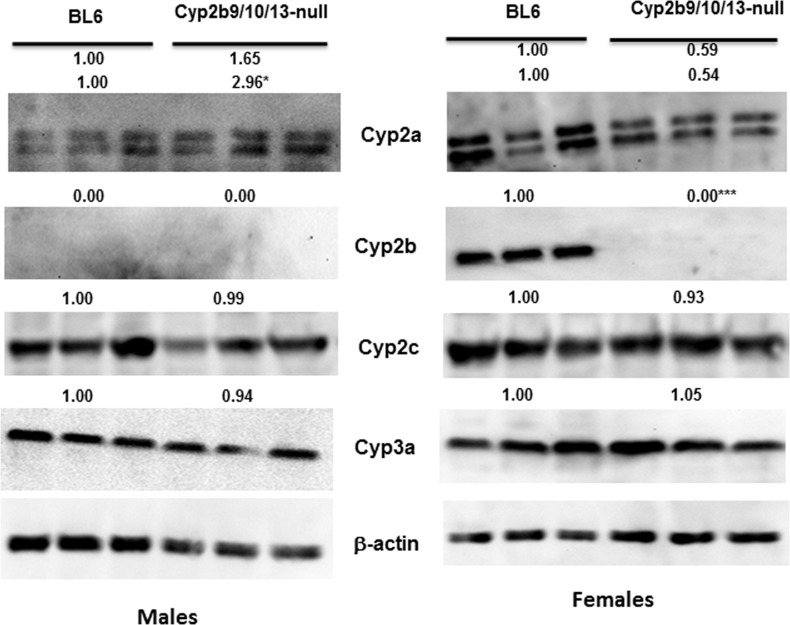
CYP protein expression in WT and Cyp2b9/10/13-null mice. Western blots of male and female Cyp2b9/10/13-null mice show significant changes in CYP expression relative to their WT counterparts. Cyp2a isoforms show two bands as Cyp2a22 is 50kDa in B6 mice and the other Cyp2a isoforms are 56 kDa. Results are expressed as relative mean of the WT compared to CAR-null mice of the same sex. Statistical differences were determined by Student’s t-tests (n = 3) with * (p < 0.05) *** (p < 0.001) indicating significant differences.

Western blots confirm the null genotype of the hepatic CYP2B proteins (Fig 6). Cyp2b is expressed at low levels in males and was not detectable in our western blots, but is clearly deleted in the Cyp2b9/10/13-null females. Western blots also confirm the down-regulation of Cyp2a mRNA in females shown by qPCR. Protein levels of Cyp2a genes dropped 41–46% in female Cyp2b9/10/13-null mice and protein levels of Cyp2a genes increased significantly (about 3-fold) in male Cyp2b9/10/13-null mice (Fig 6). This new Cyp2a antibody recognizes two bands in our Western blots unlike first antibody used with the CAR-null and Cyp3a-null mice. B6 mice have several Cyp2a isoforms and Cyp2a22, which is primarily hepatic [39, 42] is 50kDa in C57Bl6/J mice (XP_006539922.1) while the other Cyp2a isoforms (4/5/12) are 56 kDa. Messenger RNA levels of Cyp2a4 were not significantly altered in males, but Cyp2a4 mRNA increased 5-fold (Table 3), consistent with the increase in protein expression. Western blots did not confirm decreases in Cyp2c40 or Cyp3a13 protein expression in females; however, there are many Cyp2c and Cyp3a genes. Many of these other CYP isoforms were either not significantly changed, not tested, or in the case of the Cyp2c subtypes showed opposing trends (Table 3). It is also possible that the antibodies preferentially recognize specific isoforms that are not differentially expressed.

Global gene expression was measured in the livers of the null mice and compared to that in wild-type mice because there are so few compensatory changes in the Cyp2b9/10/13-null mice. Microarray data showed that in males, there are no statistically significant differentially expressed genes between the Cyp2b-null mice and wild-type mice consistent with the qPCR data. In female mice, Cyp2b9, Cyp2b10, and Cyp2b13 were all significantly down-regulated in the Cyp2b9/10/13-null strain. Cyp2a4 and Cyp2c40 were both significantly down-regulated in the female Cyp2b9/10/13-null strain compared to wild type females by qPCR. These genes were not detected as differentially expressed by microarray, possibly because probe sets for both genes are not isoform specific. Probe ID 142230_s_at targets both Cyp2a4 and 2a5, and Probe ID 1423244_at targets Cyp2c40 and Cyp2c68. Additionally, 22 probe sets corresponding to 18 genes were also differentially expressed (Table 4). Importantly, the microarrays show no detectable compensatory increases in expression of other CYP genes. Overall, there are very few compensatory changes in the Cyp2b9/10/13-null mice.

**Table 4:** Genes differentially expressed in Cyp2b9/10/13-null female mice compared to WT female mice following microarray analysis.

Gene Symbol	RefSeq Transcript ID	p-value	Fold-Change
Cyp2b10	NM_009999	2.38E-06	-13.1906
Cyp2b9	NM_010000	1.49E-10	-49.0951
AI132709	AI132709	2.80E-10	-43.2901
Dbp	NM_016974	2.27E-08	15.2984
Rnf170	NM_029965	1.45E-06	-7.01792
Lgalsl	NM_173752	3.46E-06	-1.77298
Prpf38b	NM_025845	2.92E-06	-1.42665
C77080	NM_001033189	1.14E-05	1.52239
Prlr	NM_001253781	1.71E-05	-1.59243
Dnmt3b	NM_001003960	3.07E-06	-1.8115
Sf1	NM_001110791	2.07E-05	1.3156
Pa2g4	NM_011119	4.66E-06	1.59872
Tef	NM_017376	1.19E-05	2.43846
Gstm3	NM_010359	9.57E-06	1.65659
Nr1d2	NM_011584	9.22E-06	2.49315
Slc25a37	NM_026331	2.56E-05	1.63321
Aldh1a7	NM_011921	1.93E-05	1.2603
Inpp5f	NM_178641	2.30E-05	-1.37761
Iqgap1	NM_016721	1.78E-05	-1.51826
Foxq1	NM_008239	2.36E-05	2.43071

**Fig 7:**
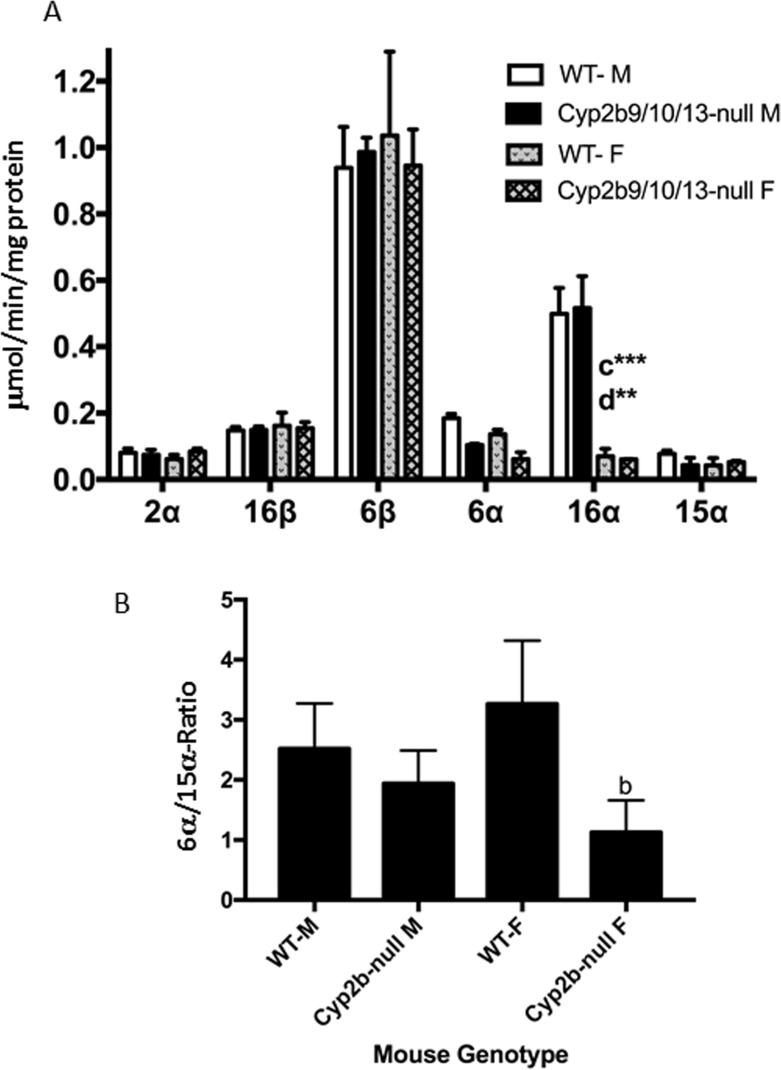
Testosterone hydroxylation determined in WT and Cyp2b9/10/13-null mice. **(A)** Testosterone hydroxylation was determined in male and female WT and Cyp2b9/10/13-null mice as described in the Materials and Methods. Data are presented as mean specific activity (μmol/min/mg protein) ± SEM (n = 4). **(B)** Ratio of 6α/15α-hydroxytestosterone as a biomarker of CYP sexual dimorphism in the liver. An ^a^indicates a significant difference between WT male and Cyp2b9/10/13-null male mice, ^b^indicates a significant difference between WT female and Cyp2b9/10/13-null female mice, ^c^indicates a significant difference between male and female WT mice and ^d^indicates a significant difference between the male and female Cyp2b9/10/13-null mice. Statistical differences were determined by two-way ANOVA followed by Fisher’s LSD as the post-hoc test in (A) and one-way ANOVA followed by Fisher’s LSD in (B). A letter without an asterisk indicates a significance of p < 0.05, asterisk indicate significance of *p<0.01, **p<0.001, and *** p<0.0001, respectively.

Testosterone hydroxylation activity did not show any significant changes in the triple knockout mice except for the expected drop in testosterone 16α-hydroxylase activity in female mice compared to male mice (Fig 7). Surprisingly, no significant changes were observed in 16β-hydroxytestosterone levels in Cyp2b9/10/13-null mice (Fig 7). Cyp2b subfamily members are known to hydroxylate at the 16α‐ and 16β-positions [60, 82, 83]. Phenobarbital and TCPOBOP, powerful CAR activators are known to induce both 16α- and 16β-hydroxylase activity, especially 16β-hydroxylase activity in part because of its induction of Cyp2b10 [16, 83]. CYP2B, CYP2C and CYP2D members contribute to 16-position hydroxylation of testosterone [16, 84, 85]; however, Cyp2d9 is the only male specific 16-hydroxylase indicating that this CYP is the primary 16α- and 16β-OH in males [16]. Therefore, our data suggests that CYP2B is not the primary 16-hydroxylase; instead it is the inducible 16-hydroxylase [16, 43, 86].

Interestingly, we also observed a significant (p<0.01) reduction in 6α-OH testosterone activity in the Cyp2b9/10/13-null mice compared to their WT counterparts using one-way ANOVA followed by Fisher’s LSD post-hoc (p < 0.05) instead of the two-way ANOVA showed in the figure. This either suggests that Cyp2b members are involved in the constitutive metabolism of testosterone in the 6α-position or that there is a drop in Cyp2a members crucial in 6α-hydroxylation. Testosterone 6α-hydroxylase activity was also decreased in CAR-null mice, but not Cyp3a-null mice (Fig 3). A drop in Cyp2a was measured in Cyp2b9/10/13-null females but not males (Fig 6; Table 3). In turn, 6α/15α ratio was significantly higher in WT females than Cyp2b9/10/13-null females (2.6X), suggesting weak to moderate hepatic masculinization of Cyp2b9/10/13-null females. There were no differences in serum or hepatic testosterone concentrations between WT and Cyp2b9/10/13-null mice indicating that the difference in 6α/15α ratio is directly due to the drop in Cyp2a protein expression or loss of Cyp2b’s.

## Conclusions

CAR-null mice show greater changes in CYP expression and activity relative to their WT counterparts than Cyp3a-null and Cyp2b-null mice. This is probably because CAR directly regulates the expression of several CYPs either constitutively or through activation by endogenous and exogenous substrates. Because CAR regulates constitutive CYP expression the use of CAR-null mice alone could cause incorrect interpretations of chemical metabolism. In addition, CAR appears to regulate sexual dimorphism of CYP expression within the liver as lack of CAR caused masculinization. Given that CAR activation is feminizing [16, 43, 67], CAR has greater transcriptional activity in females than males [50], CAR regulates a number of female predominant CYPs [16], and CAR is inhibited by androgens [11, 53], it may not be all that surprising that the loss of CAR causes masculinization of the liver (Fig 3). Overall, CAR-null mice may show significant changes in CYP-mediated drug metabolism following exposure because of the significant changes in CYP expression.

Cyp3a-null mice show some compensatory changes in CYP expression and testosterone metabolism. We would not expect the changes to be as broad as the CAR-null mice because the regulation of the other CYPs is not direct. However, the small changes observed in 2α-OH and 16α-OH testosterone levels suggest that the loss of CYP3A activity alters liver substrate profiles for CAR, PXR, and potentially other nuclear receptors/transcription factors that regulate CYP expression. Alternatively, several CYPs may show changes in CYP activity because they compensate for the loss of CYP3A and the lack of competition for the substrate. Based on the changes in expression profiles such as significant increases in Cyp2a4 and Cyp3a13 (the only Cyp3a gene retained in the knockout mouse model), we would predict that the lack of CYP3A is causing the activation of PXR and potentially a drop in CAR activity. Additionally, the observed increase, though not significant, in expression of Cyp2b10, Cyp2c29 and Cyp2c40 suggest increase in PXR activity in Cyp3a-null mice.

Cyp2b-null mice show very few overall changes in CYP expression outside the loss of CYP2B (Tables 3 and 4; Figs 6 and 7). Unlike CYP3A, which is the most abundant CYP in murine livers, CYP2B shows relatively low expression, lower than CYP3A, 2C, and 2D subfamily members [24]. Therefore, we would expect fewer compensatory changes. Overall, most if not all changes in drug metabolism in the Cyp2b9/10/13-null mice would reflect the loss of Cyp2b.

In conclusion, toxicology models such as CAR-null and various CYP-null mice show changes in other CYPs due to direct control of expression or compensatory changes in expression. These changes in CYP expression, especially those regulated by CAR, may alter hepatic CYP expression, CYP-mediated xenobiotic metabolism, and hepatic CYP masculinization. Perturbations in non-CYP3A/2B-mediated metabolism of xenobiotics may occur in these models with significant changes in metabolism more likely in CAR-null compared to Cyp3a-null or Cyp2b-null mice.

## Supporting information

S1 Fig A cluster of Cyp2b genes are found on chromosome 7 (26,500K – 27,630K). All five Cyp2b subfamily members are located in the 7A region of chromosome 7. However, there are six genes between two Cyp2b regions; Therefore, we knocked out the three predominant hepatic CYPs (Cyp2b9/10/13) via partial chromosomal deletion using Crispr/Cas9 because it would not impact other genes.
(PDF)

S2 Fig Testosterone concentrations in nullizygous mice. Testosterone concentrations in the liver of CAR-null (A), Cyp3a-null (B), Cyp2b9/10/13-null (C) and serum of Cyp2b9/10/13-null mice were measured and compared to their WT counterparts. Testosterone concentrations from liver cytosol or serum were measured by EIA using a kit from Cayman Chemical Company (Ann Arbor, MI). Data are presented as mean testosterone concentrations ± SEM (n = 3–4). A ^c^indicates a significant difference between male and female WT mice and ^d^indicates a significant difference between male and female nullizygous mice. There are no significant differences between nullizygous mice and their WT counterparts. Statistical differences were determined by one-way ANOVA followed by Fisher’s LSD as the post-hoc test A letter without an asterisk indicates a significance of p < 0.05, asterisk indicate significance of **p<0.001, and *** p<0.0001, respectively.
(PDF)
