# Supplementary material for: Compensatory changes in CYP expression in three different toxicology mouse models: CAR-null, Cyp3a-null, and Cyp2b9/10/13-null mice
Source: PLoS One. 2017 Mar 28;12(3):e0174355. doi: 10.1371/journal.pone.0174355 (PMC5370058; doi:10.1371/journal.pone.0174355)
Supplement: S1 Fig — All five Cyp2b subfamily members are located in the 7A region of chromosome 7. However, there are six genes between two Cyp2b regions; Therefore, we knocked out the three predominant hepatic CYPs (Cyp2b9/10/13) via partial chromosomal deletion using Crispr/Cas9 because it would not impact other genes. (PDF) [file pone.0174355.s001.pdf]

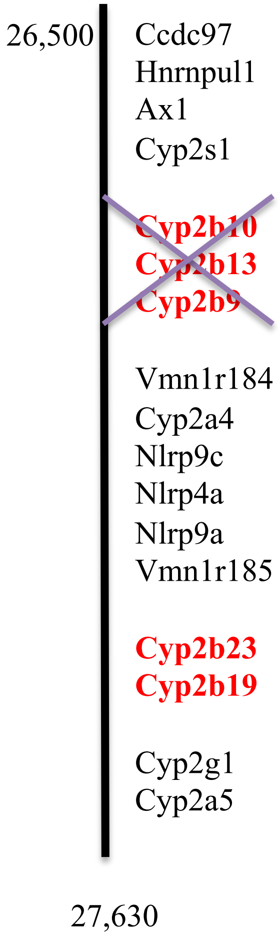

S1 Fig. A cluster of Cyp2b genes are found on chromosome 7 (26,500K – 27,630K). All five Cyp2b subfamily members are located in the 7A region of chromosome 7. However, there are six genes between two Cyp2b regions; Therefore, we knocked out the three predominant hepatic CYPs (Cyp2b9/10/13) via partial chromosomal deletion using Crispr/Cas9 because it would not impact other genes.
