# Supplementary material for: Compensatory changes in CYP expression in three different toxicology mouse models: CAR-null, Cyp3a-null, and Cyp2b9/10/13-null mice
Source: PLoS One. 2017 Mar 28;12(3):e0174355. doi: 10.1371/journal.pone.0174355 (PMC5370058; doi:10.1371/journal.pone.0174355)

S2 Fig. Testosterone concentrations in nullizygous mice. Testosterone concentrations in the liver of CAR-null (A), Cyp3a-null (B), Cyp2b9/10/13-null (C) and serum of Cyp2b9/10/13-null mice were measured and compared to their WT counterparts. Testosterone concentrations from liver cytosol or serum were measured by EIA using a kit from Cayman Chemical Company (Ann Arbor, MI). Data are presented as mean testosterone concentrations ± SEM (n = 3-4). A [c]indicates a significant difference between male and female WT mice and [d]indicates a significant difference between male and female nullizygous mice. There are no significant differences between nullizygous mice and their WT counterparts. Statistical differences were determined by one-way ANOVA followed by Fisher's LSD as the post-hoc test A letter without an asterisk indicates a significance of $p < 0.05$, asterisk indicate significance of $^{**}p<0.001$, and $^{***}p<0.0001$, respectively.

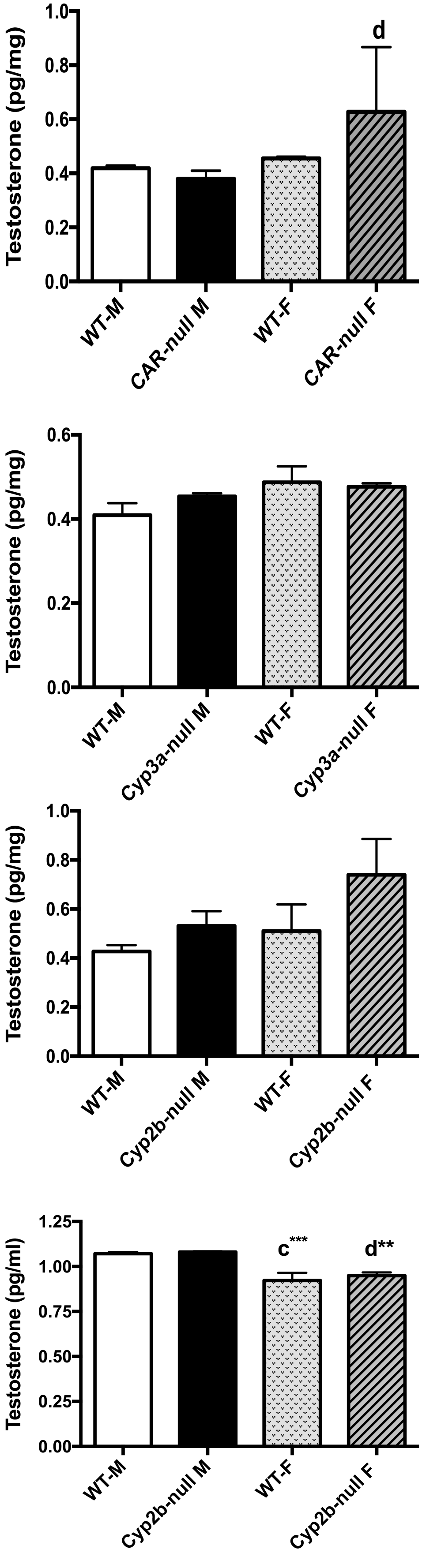

Supplement: S2 Fig — Testosterone concentrations in the liver of CAR-null (A), Cyp3a-null (B), Cyp2b9/10/13-null (C) and serum of Cyp2b9/10/13-null mice were measured and compared to their WT counterparts. Testosterone concentrations from liver cytosol or serum were measured by EIA using a kit from Cayman Chemical Company (Ann Arbor, MI). Data are presented as mean testosterone concentrations ± SEM (n = 3–4). A cindicates a significant difference between male and female WT mice and dindicates a significant difference between male and female nullizygous mice. There are no significant differences between nullizygous mice and their WT counterparts. Statistical differences were determined by one-way ANOVA followed by Fisher’s LSD as the post-hoc test A letter without an asterisk indicates a significance of p < 0.05, asterisk indicate significance of **p<0.001, and *** p<0.0001, respectively. (PDF) [file pone.0174355.s002.pdf]
